# Antimicrobial Resistance of *Salmonella enterica* Serovar Typhimurium in Shanghai, China

**DOI:** 10.3389/fmicb.2017.00510

**Published:** 2017-03-28

**Authors:** Jinyan Wang, Yongrui Li, Xuebin Xu, Beibei Liang, Fuli Wu, Xiaoxia Yang, Qiuxia Ma, Chaojie Yang, Xiaofeng Hu, Hongbo Liu, Hao Li, Chunyu Sheng, Jing Xie, Xinying Du, Rongzhang Hao, Shaofu Qiu, Hongbin Song

**Affiliations:** ^1^Institute of Disease Control and Prevention, Academy of Military Medical SciencesBeijing, China; ^2^Western Theater CommandTianshui, China; ^3^The Key laboratory of Pharmacology and Molecular Biology, Medical College, Henan University of Science and TechnologyLuoyang, China; ^4^Shanghai Municipal Center for Disease Control and PreventionShanghai, China

**Keywords:** *Salmonella*, ESBL, ciprofloxacin, azithromycin, PMQR

## Abstract

We aimed to analyze the antimicrobial resistance phenotypes and to elucidate the molecular mechanisms underlying resistance to cephalosporins, ciprofloxacin, and azithromycin in *Salmonella enterica* serovar Typhimurium isolates identified from patients with diarrhea in Shanghai. The isolates showed high rates of resistance to traditional antimicrobials, and 20.6, 12.7, and 5.5% of them exhibited decreased susceptibility to cephalosporins, ciprofloxacin, and azithromycin, respectively. Notably, 473 (84.6%) isolates exhibited multidrug resistance (MDR), including 161 (28.8%) isolates that showed an ACSSuT profile. Twenty-two MDR isolates concurrently exhibited decreased susceptibility to cephalosporins and ciprofloxacin, and six of them were co-resistant to azithromycin. Of all the 71 isolates with decreased susceptibility to ciprofloxacin, 65 showed at least one mutation (D87Y, D87N, or D87G) in *gyrA*, among which seven isolates simultaneously had mutations of *parC* (S80R) (*n* = 6) or *parC* (T57S/S80R) (*n* = 1), while 49 isolates with either zero or one mutation in *gyrA* contained plasmid-mediated quinolone resistance (PMQR) genes including *qnrB, qnrS, and aac(6′)-Ib-cr*. Among the 115 cephalosporin-resistant isolates, the most common ESBL gene was *bla*_CTX-M_, followed by *bla*_TEM-1_, *bla*_OXA-1_, and *bla*_SHV -12_. Eight subtypes of *bla*_CTX-M_ were identified and *bla*_CTX-M-14_ (*n* = 22) and *bla*_CTX-M-55_ (*n* = 31) were found to be dominant. To the best of our knowledge, this is the first report of the presence of *bla*_CTX-M-123_ and *bla*_CTX-M-125_ in *S.* Typhimurium. Besides, *mphA* gene was identified in 15 of the 31 azithromycin-resistant isolates. Among the 22 isolates with reduced susceptibility to cephalosporins and ciprofloxacin, 15 contained ESBL and PMQR genes. Coexistence of these genes lead to the emergence of MDR and the transmission of them will pose great difficulties in *S.* Typhimurium treatments. Therefore, surveillance for these MDR isolates should be enhanced.

## Introduction

*Salmonella* infection is a major global public health problem, which has caused food-borne illnesses in many countries. Data from the Foodborne Diseases Active Surveillance Network (Foodnet) report showed that *Salmonella* has become a leading cause of death as a food-borne bacterial pathogens in the United States (Barton Behravesh et al., 2011). Among the more than 2500 serotypes of *Salmonella* (Popoff et al., 2001), *S.* Typhimurium is one of the predominant serotypes in many developed and developing countries, and the global outbreak of food-borne diseases due to infection by *S*. Typhimurium is impressive. *S.* Typhimurium infection was very common in the United States; for example, *S.* Typhimurium enteritis had been diagnosed in 600 persons in 44 states due to peanut butter contamination in 2008 (Maki, 2009). *S.* Typhimurium infection has also been often reported in China and was found to be the second most prevalent serotype (Ran et al., 2011).

Antimicrobial treatment is needed for infants, the elderly, and immunocompromised individuals with *Salmonella* infection. Fluoroquinolones and cephalosporins are the primary choice for clinical treatment (Glynn et al., 1998). In addition, azithromycin has gained the approval of the Food and Drug Administration (FDA) as an additional agent to treat *Salmonella* infections (Sjolund-Karlsson et al., 2011). With the extensive use of such antimicrobials, antimicrobial resistance is increasing at a serious rate in *Salmonella* isolates. In many countries, more and more *Salmonella* strains with MDR (defined as resistance to three or more classes of antimicrobials) have been discovered since the 1990’s report of the spread of MDR in *S.* Typhimurium of definitive phage type 104 (DT104) in the world (Molbak et al., 1999). Relevant monitoring data showed that MDR in the entire *Salmonella* genus has increased from 20 to 30% in the early 1990s to 70% at the beginning of this century (Su et al., 2004). *S.* Typhimurium has also been found to show high rates of resistance to the traditional antimicrobials, and resistance to ciprofloxacin or cephalosporins have been found to have emerged in countries such as France and the United States (Weill et al., 2006; Whichard et al., 2007). A mass of *S.* Typhimurium isolates were also found to be resistant to ciprofloxacin or cephalosporins in many cities of China (Cui et al., 2008; Jiang et al., 2014). In recent years, some cases of azithromycin- resistance in patients with *Salmonella* infection were reported (Sjolund-Karlsson et al., 2011). Surprisingly, two strains have even been reported to be concurrently resistant to ciprofloxacin, cephalosporins, and azithromycin in China (Wong et al., 2014).

Shanghai is an economic and financial center located in eastern China and is divided into 16 administrative units with more than 24 million people. In order to better monitor public health issues, the Shanghai Center for Disease Control and Prevention (SCDC) became a member of the World Health Organization Global Food-borne Infections Network (WHO-GFN) in 2005 (Zhang et al., 2014). In this study, we aimed to analyze the antimicrobial resistance profiles of *S.* Typhimurium in Shanghai from 2011 to 2014 and tried to elucidate the molecular mechanisms underlying the emergence of MDR in these isolates. Our findings highlight the importance of increasing surveillance on these isolates with MDR, which will help provide appropriate clinical antimicrobial treatment for patients with *S.* Typhimurium infection.

## Materials and Methods

### Specimen Collection and Isolate Identification

Fresh stool samples from clinically suspected patients who had diarrhea were collected from 24 sentinel hospitals and eight regional SCDC diagnostic laboratories in Shanghai as described previously (Zhang et al., 2014). The stool samples were then enriched in Selenite Brilliant Green broth (CHROMagar, China) for 16–22 h at 37°C. The enriched samples were then cultivated on *Salmonella-Shigella* (SS) agar or xylose lylose deoxycholate agar (XLD; CHROMagar, China) and incubated for 18–24 h at 37°C. Presumptive colonies were screened by testing in triple-sugar-iron agar, motility indole urea agar, L-lysine decarboxylase, and L-galactosidase (*o*-nitrophenyl-L-D-galactopyranoside; ONPG). One presumptive colony from each sample was stored in semisolid agar and sent to our laboratory for further confirmation. API 20E test strips (bioMerieux Vitek, Marcy-l’Etoile, France) were used to confirm the identity of the isolates. All the isolates were then serotyped by slide agglutination with commercial antiserum (S&A Reagents Laboratory, Thailand) according to the Kauffmann-White scheme (World Health Organization, 2011). This study was approved by Institute of Disease Control and Prevention, Academy of Military Medical Sciences (Beijing, China) and the informed consent was obtained from the subjects involved in this study.

### Antimicrobial Susceptibility Testing

The minimum inhibitory concentrations (MICs) for 14 antimicrobial agents including cefoxitin (FOX), ceftriaxone (CRO), ceftiofur (XNL), azithromycin (AZI), chloramphenicol (CHL), tetracycline (TET), ciprofloxacin (CIP), gentamicin (GEN), nalidixic acid (NAL), sulfisoxazole (FIS), ampicillin (AMP), streptomycin (STR), amoxicillin/clavulanic acid at a 2:1 ratio (AUG2), and trimethoprim/sulfamethoxazole (SXT) were evaluated by broth microdilution using the Sensititre plate CMV3AGNF (Sensititre, Thermo Fisher Scientific, USA) according to the recommendations of the Clinical and Laboratory Standards Institute (CLSI) (Clinical Laboratory Standards Institute, 2016). An *Escherichia coli* ATCC 25922 strain was used for quality control.

### PCR Amplification and DNA Sequencing

All the cephalosporin-resistant isolates were analyzed using PCR assays for the presence of extended-spectrum β-lactamase (ESBL) genes such as *bla*_CTX-M_ groups, *bla*_OXA_, *bla*_TEM_, *bla*_SHV_, and *bla*_CMY_ (Hasman et al., 2005; Weill et al., 2006; Cui et al., 2015). PCR amplification of quinolone resistance-determining regions (QRDRs) of *gyrA, gyrB, parC*, and *parE* (Giraud et al., 1999) and plasmid-mediated quinolone resistance (PMQR) determinants [*qnrA, qnrB, qnrD, qnrS*, and *aac(6′)-Ib-cr*] (Park et al., 2006; Cui et al., 2015) were performed on the ciprofloxacin-resistant or intermediate resistant isolates. All azithromycin-resistant isolates were subjected to PCR to detect macrolide-resistance genes including *mphA, mphB, ermA, ermB, ereA, mefA*, and *msrA* (Phuc Nguyen et al., 2009). The primers used for the above mentioned PCR amplifications are shown in **Table 1**. All the PCR products were sequenced by the Beijing Genomics Institute (BGI). Sequences data were then analyzed by DNAstar (DNAstar Inc., Madison, WI, USA) and the sequences were compared with reference sequences from NCBI GenBank.

**Table 1:** Primers for the PCR detection of antimicrobal-resistance determinants.

Target	Primer sequence (5′–3′)	Amplicon size (bp)	Reference
**β-Lactamases**			
*bla*_CTX-M-1_ group	F:GGTTAAAAAATCACTGCGTC	873	Cui et al., 2015
	R:TTACAAACCGTCGGTGACGA		
*bla*_CTX-M-9_ group	F:AGAGTGCAACGGATGATG	868	Cui et al., 2015
	R:CCAGTTACAGCCCTTCGG		
*bla*_CTX-M-2/8/25_ group	F:ACCGAGCCSACGCTCAA	221	Cui et al., 2015
	R:CCGCTGCCGGTTTTATC		
*bla*_OXA-1_ group	F:ATGAAAAACACAATACATATC	830	Weill et al., 2006
	R:AATTTAGTGTGTTTAGAATGG		
*bla*_TEM_	F:ATAAAATTCTTGAAGACGAAA	1080	Weill et al., 2006
	R:GACAGTTACCAATGCTTAATC		
*bla*_SHV_	F:TTATCTCCCTGTTAGCCACC	795	Weill et al., 2006
	R:GATTTGCTGATTTCGCTCGG		
*bla*_CMY_	F:GTGGTGGATGCCAGCATCC	915	Hasman et al., 2005
	R:GGTCGAGCCGGTCTTGTTGAA		
**QRDR of Topoisomerase genes**			
*gyrA*	F: TGGGCAATGACTGGAACA	431	This study
	R: GGTTGTGCGGCGGGATA		
*gyrB*	F: ATGAGCGATATGGCAGAGCG	309	Cui et al., 2015
	R:GCTGTGATAACGCAGTTTGTCCGGG		
*parC*	F:ATGAGCGATATGGCAGAGCG	413	Giraud et al., 1999
	R:TGACCGAGTTCGCTTAACAG		
*parE*	F:ATGCGTGCGGCTAAAAAAGTG	290	Cui et al., 2015
	R:TCGTCGCTGTCAGGATCGATAC		
**PMQR**			
*qnrA*	F:ATTTCTCACGCCAGGATTTG	516	Cui et al., 2015
	R:GATCGGCAAAGGTTAGGTCA		
*qnrB*	F:GATCGTGAAAGCCAGAAAGG	469	Cui et al., 2015
	R:ACGATGCCTGGTAGTTGTCC		
*qnrD*	F:CGAGATCAATTTACGGGGAATA	656	Cui et al., 2015
	R:AACAAGCTGAAGCGCCTG		
*qnrS*	F:ACGACATTCGTCAACTGCAA	417	Cui et al., 2015
	R:TAAATTGGCACCCTGTAGGC		
*aac(6′)-Ib-cr*	F:TTGCGATGCTCTATGAGTGGCTA	482	Park et al., 2006
	R:CTCGAATGCCTGGCGTGTTT		
**Macrolide-resistance Genes**			
*mphA*	F:GTGAGGAGGAGCTTCGCGAG	403	Phuc Nguyen et al., 2009
	R:TGCCGCAGGACTCGGAGGTC		
*mphB*	F:GATATTAAACAAGTAATCAGAATAG	494	Phuc Nguyen et al., 2009
	R:GCTCTTACTGCATCCATACG		
*ermA*	F:TCTAAAAAGCATGTAAAAGAAA	533	Phuc Nguyen et al., 2009
	R:CGATACTTTTTGTAGTCCTTC		
*ermB*	F:GAAAAAGTACTCAACCAAATA	693	Phuc Nguyen et al., 2009
	R:AATTTAAGTACCGTTACT		
*ereA*	F:GCCGGTGCTCATGAACTTGAG	420	Phuc Nguyen et al., 2009
	R:CGACTCTATTCGATCAGAGGC		
*mefA*	F:AGTATCATTAATCACTAGTGC	345	Phuc Nguyen et al., 2009
	R:TTCTTCTGGTACTAAAAGTGG		
*msrA*	F:GCACTTATTGGGGGTAATGG	384	Phuc Nguyen et al., 2009
	R:GTCTATAAGTGCTCTATCGTG		

### Statistical Analysis

Chi-squared test was used for data analysis using SPSS software (SPSS Inc., Chicago, IL, USA; version 17.0); a *P-*value < 0.05 was considered to indicate statistical significance.

## Results

### *S.* Typhimurium Isolates from Human Patients in Shanghai, China, from 2011 to 2014

**FIGURE 1:**
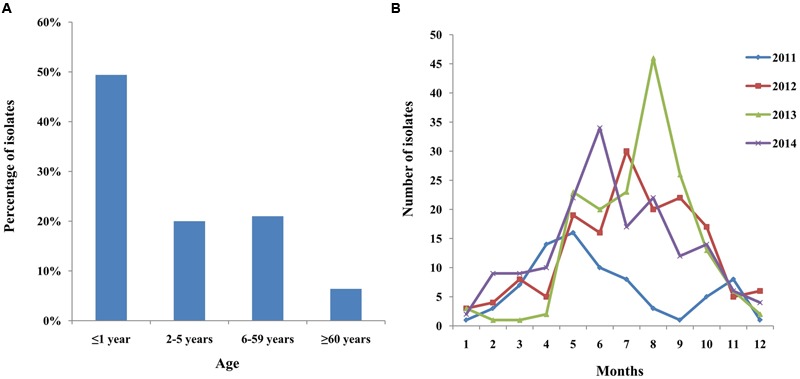
**Distribution of human *S.* Typhimurium isolates in patients by (A)** age and **(B)** months (2011–2014) in Shanghai, China.

Between January 2011 and December 2014, 559 *S.* Typhimurium isolates were cultured from patients with diarrhea in Shanghai, China. The age of the patients ranged from 10 days to 86 years (19 cases unknown) (**Figure 1**). Children under 5 years of ages, and especially less than 1-year-old children, were highly susceptible to *S.* Typhimurium infection, which accounted for 49.4% of all the patients (*P* < 0.05). The ratio of the male to female patients was 1.3:1. Infections occurred mainly in summer and autumn (**Figure 1**).

### Antimicrobial Susceptibility Testing

Among the 559 isolates, only 26 (4.7%) were susceptible to all 14 antimicrobials. Resistance to tetracycline was the most common (83.7%), followed by ampicillin (80.1%), nalidixic acid (79.2%), sulfisoxazole (77.1%), chloramphenicol (54.9%), streptomycin (53.7%), trimethoprim/sulfamethoxazole (47.4%), gentamicin (37%), and amoxicillin/clavulanic acid (7.9%) (**Table 2**). In addition, resistance to ceftiofur, ceftriaxone, and cefoxitin was found in 20, 20, and 5% of isolates, respectively. Notably, 3.6% (20/599) of the isolates displayed resistance (MIC ≥ 4 μg/mL) to ciprofloxacin, and 9.1% (51/599) exhibited intermediate resistance to ciprofloxacin (MICs = 2 μg/mL). More importantly, 22 of the isolates showed co-resistance to cephalosporins. Moreover, 31 (5.5%) isolates were showed resistance to azithromycin, and 6 of them were co-resistant to cephalosporins and ciprofloxacin.

**Table 2:** Antimicrobial resistance of *S.* Typhimurium isolates in Shanghai from 2011 to 2014.

	*N* (%) of resistant isolates				
	2011 (*N* = 77)	2012 (*N* = 155)	2013 (*N* = 167)	2014 (*N* = 160)	Total (*N* = 559)
**Pan-susceptible**	4 (5.2)	8 (5.2)	4 (2.4)	10 (6.3)	26 (4.7)
Nalidixic Acid	67 (87)	107 (60)	145 (86.8)	124 (77.5)	443 (79.2)
Ciprofloxacin	7 (9.1)	4 (2.6)	6 (3.6)	3 (1.9)	20 (3.6)
Ampicillin	54 (70.1)	124 (80)	150 (89.8)	120 (75)	448 (80.1)
Ceftiofur	23 (29.9)	37 (23.9)	30 (18)	22 (13.8)	112 (20)
Cefoxitin	8 (10.4)	17 (10.3)	1 (0.6)	2 (3.8)	28 (5)
Ceftriaxone	23 (29.9)	37 (23.9)	30 (18)	22 (13.8)	112 (20)
Tetracycline	58 (75.3)	124 (80)	156 (93.4)	130 (81.3)	468 (83.7)
Streptomycin	33 (42.9)	74 (47.7)	97 (58.1)	96 (60)	300 (53.7)
Gentamicin	29 (37.7)	62 (40)	81 (48.5)	35 (21.9)	207 (37)
Chloramphenicol	31 (40.3)	86 (55.5)	124 (74.3)	66 (41.3)	307 (54.9)
Azithromycin	1 (1.3)	13 (8.4)	4 (2.4)	13 (8.1)	31 (5.5)
Sulfisoxazole	27 (35.1)	126 (81.3)	156 (93.4)	122 (76.3)	431 (77.1)
trimethoprim/sulfamethoxazole	31 (40.3)	76 (49)	97 (58.1)	61 (38.1)	265 (47.4)
amox-icillin/clavulanic acid	9 (11.7)	20 (12.9)	5 (3)	10 (6.3)	44 (7.9)
**MDR pattern**					
≥3 antimicrobials	56 (72.7)	130 (83.9)	159 (95.2)	128 (80)	473 (84.6)
≥4 antimicrobials	51 (66.2)	121 (78.1)	147 (88)	116 (72.5)	435 (77.8)
≥5 antimicrobials	40 (51.9)	91 (58.7)	125 (74.9)	85 (53.1)	341 (61)
≥6 antimicrobials	25 (32.5)	52 (33.5)	91 (54.5)	47(29.4)	215 (38.5)
≥7 antimicrobials	1 (1.3)	0 (0)	1 (0.6)	4 (2.5)	6 (1.1)
ACSSuT	15 (19.5)	42 (27.1)	66 (39.5)	38 (23.8)	161 (28.8)
ACSSuT+CIP	5 (6.5)	6 (3.9)	18 (10.8)	11 (6.9)	40 (7.2)
ACSSuT+CEP	7 (9.1)	15 (9.7)	8 (4.8)	6 (3.8)	36 (6.4)
ACSSuT+AZI	1 (1.3)	2 (1.3)	1 (0.6)	6 (3.8)	10 (1.8)
ACSSuT+CIP+CEP	2 (2.6)	0 (0)	2 (1.2)	2 (1.3)	6 (1.1)
ACSSuT+CIP+CEP+AZI	1 (0.2)	0 (0)	0 (0)	0 (0)	1 (0.2)

*CIP, Ciprofloxacin; CEP, cephalosporins; AZI, Azithromycin.*

Multidrug resistance was observed in 84.6% of the isolates, in which ≥3, ≥4, ≥5, ≥6, and ≥7 classes of antimicrobials were found in 84.6, 77.8, 61, 38.5, and 1.1% of the MDR isolates, respectively. Among the MDR isolates, 161 (28.8%) showed the ACSSuT resistance pattern (defined as resistance to ampicillin, chloramphenicol, streptomycin, sulfamethoxazole, and tetracycline); among them, 40 (7.2%) isolates exhibited reduced susceptibility to ciprofloxacin, 36 (6.4%) were resistant to cephalosporins, and 10 (1.8%) were resistant to azithromycin. It is noteworthy that 6 (1.1%) isolates with ACSSuT resistance pattern were concurrently resistant to ciprofloxacin and cephalosporins, and one of them was also co-resistant to azithromycin (**Table 2**).

### PCR Detection of Antimicrobial Drug Resistance Genes and DNA Sequencing

**FIGURE 2:**
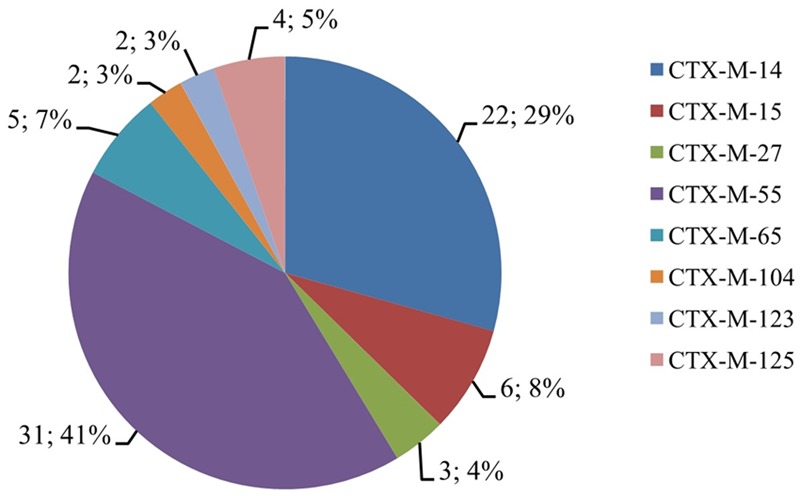
**The CTX-M proportions of cephalosporin-resistant *S.* Typhimurium isolates.** The digit before “;” indicates the number of isolates that contained *bla*_CTX-M_ genes, and the digit after “;” indicates the percentage of isolates that contained *bla*_CTX-M_ genes.

Resistance genes including *bla*_OXA_, *bla*_TEM_, *bla*_SHV_, *bla*_CMY_, *and bla*_CTX-M_ in the 115 cephalosporin-resistance isolates were detected by PCR. PCR screenings revealed that 75 (65.2%), 52 (45.2%), 43 (37.4%), and 3 (2.6%) isolates contained the *bla*_CTX_, *bla*_TEM_, *bla*_OXA_, and *bla*_SHV_ genes, respectively. All the strains were negative for *bla*_CMY_. Fifty-two isolates contained two of the above described genes (*bla*_CTX-M_+*bla*_TEM_*/bla*_CTX-M_+*bla*_OXA_*/bla*_TEM_+*bla*_SHV_), and nine isolates concurrently contained *bla*_CTX-M_, *bla*_TEM_, and *bla*_OXA_. Sequencing of the PCR products revealed that *bla*_TEM-1_, *bla*_OXA-1_, and *bla*_SHV -12_ were the main genotypes. *bla*_CTX-M_ included eight subtypes: *bla*_CTX-M-14_, *bla*_CTX-M-15_, *bla*_CTX-M-27_, *bla*_CTX-M-55_, *bla*_CTX-M-65_, *bla*_CTX-M-104_, *bla*_CTX-M-123_, and *bla*_CTX-M-125_, among which *bla*_CTX-M-14_ and *bla*_CTX-M-55_ were the most common subtypes (**Figure 2**).

We identified 71 isolates with decreased susceptibility to ciprofloxacin, and 65 of them contained at least one *gyrA* mutation which mainly occurred at encode 87 (D87Y, D87N, or D87G). Among them, six had double mutations (S83F/D87N or D87G) in the *gyrA* gene and a single mutation (S80R) in the *parC* gene; 1 isolate with four mutations, including two in *gyrA* gene (S83F/D87N) and two in *parC* gene (T57S/S80R); 58 isolates with only one *gyrA* mutation (D87Y or D87N). No point mutations in *gyrB* and *parE* were found. Here, three species of PMQR determinants including *aac(6′)-Ib-cr* (*n* = 50), *qnrB* (*qnrB2, n* = 2; *qnrB4, n* = 4), and *qnrS1* (*n* = 1) were detected. Among the 49 isolates with no mutation or only one mutation in *gyrA* gene, one or two PMQR genes were detected (**Table 3**).

**Table 3:** Distribution of PMQR genes and mutations in *gyrA, gyrB, parC*, and *parE* genes in *S.* Typhimurium isolates with decreased susceptibility to ciprofloxacin in Shanghai, China.

QRDR mutation				PMQR	*N*		Total
*gyrA*	*gyrB*	*parC*	*parE*		I^∗^	R^∗∗^	
WT	WT	WT	WT	*qnrS*	1	0	1
WT	WT	WT	WT	*aac(6*′*)-Ib-cr*	1	0	1
WT	WT	WT	WT	*aac(6*′*)-Ib-cr,qnrB*	1	3	4
D87Y	WT	WT	WT	WT	15	0	15
D87Y	WT	WT	WT	*aac(6*′*)-Ib-cr*	20	4	24
D87N	WT	WT	WT	*aac(6*′*)-Ib-cr*	13	4	17
D87Y	WT	WT	WT	*aac(6*′*)-Ib-cr,qnrB*	0	2	2
S83F/D87N	WT	S80R	WT	WT	0	4	4
S83F/D87N	WT	S80R	WT	*aac(6*′*)-Ib-cr*	0	1	1
S83F/D87G	WT	S80R	WT	WT	0	1	1
S83F/D87N	WT	T57S/S80R	WT	*aac(6*′*)-Ib-cr*	0	1	1

*^∗^*S*. Typhimurium isolates with intermediate resistance to CIP (MIC = 2 μg/mL); ^∗∗^*S*. Typhimurium isolates with resistance to CIP (MIC ≥ 4 μg/mL); WT, wild type.*

In addition, PCR results showed that 15 out of the 31 azithromycin-resistant isolates (MIC ≥ 128 μg/mL) harbored *mphA* genes, while the other 16 (MIC: 16–64 μg/mL) did not. All the azithromycin-resistant isolates were negative for *mphB, ermA, ermB, ereA, mefA*, and *msrA*.

Notably, among the 22 isolates that concurrently exhibited reduced susceptibility to cephalosporins and ciprofloxacin, 15 contained ESBL and PMQR genes. Two isolates contained four types of antimicrobial-resistance genes: *qnrB*/*aac(6*′*)-Ib-cr*/*bla*_CTX-M_/*bla*_TEM_ (*n* = 1) and *aac(6*′*)-Ib-cr/bla*_OXA_/*bla*_CTX-M_/*bla*_TEM_ (*n* = 1). Nine isolates harbored three types of antimicrobial resistant genes, including *qnrB /aac(6*′*)-Ib-cr/bla*_OXA_ (*n* = 2), *aac(6*′*)-Ib-cr/bla*_OXA_/*bla*_CTX-M_ (*n* = 4), and *aac(6*′*)-Ib-cr*/*bla*_CTX-M_/*bla*_TEM_ (*n* = 3). Four isolates contained two types of antimicrobial-resistant genes: *aac(6*′*)-Ib-cr*/*bla*_TEM_ (*n* = 2) and *aac(6*′*)-Ib-cr*/*bla*_CTX-M_ (*n* = 2). In addition, 2 out of the 15 isolates that were co-resistant to azithromycin concurrently harbored ESBL, PMQR, and *mphA* genes: *aac(6*′*)-Ib-cr*/*bla*_CTX-M_*/bla*_TEM_/*mphA* (**Table 4**).

**Table 4:** Summary of phenotypes of *S.* Typhimurium isolates showing concurrently decreased susceptibility to ciprofloxacin and cephalosporins and their corresponding resistance genes.

Strain	MIC (μg/mL)					QRDR mutation		PMQR	ESBL	*mphA*
	FOX	CRO	XNL	CIP	AZI	*gyrA*	*parC*			
SHI 1G663	>32	4	8	4	4	WT	WT	*qnrB/aac(6*′*)-Ib-cr*		
SHI 1G664	>32	8	>8	4	2	WT	WT	*qnrB/aac(6*′*)-Ib-cr*	*bla*_OXA_	
SHI 1G665	>32	32	8	4	4	WT	WT	*qnrB/aac(6*′*)-Ib-cr*	*bla*_OXA_	
SH11G680	4	>64	>8	>4	2	S83F/D87N	S80R		*bla*_OXA_*/bla*_CTX-M-55_	
SHI 1G993	>32	16	>8	>4	256	S83F/D87N	S80R		*bla*_OXA_	*mphA*
SH11G1304	2	>64	>8	2	4	D87Y	WT	*aac(6*′*)-Ib-cr*	*bla*_OXA_/bla_CTX-M-27_	
SH11G1306	2	>64	>8	2	4	D87Y	WT			
SHI 2G734	32	<0.25	2	2	4	D87Y	WT	*aac(6*′*)-Ib-cr*	*bla*_TEM_	
SHI 2G889	32	<0.25	2	4	8	D87Y	WT	*aac(6*′*)-Ib-cr*	*bla*_OXA_*/bla*_CTX-M-14_*/bla*_TEM_	
SH12G978	32	>64	>8	2	64	WT	WT	*qnrB/aac(6*′*)-Ib-cr*	*bla*_CTX-M-15_*/bla*_TEM_	
SHI 2G1005	8	>64	>8	>4	64	S83F/D87N	T57S/S80R	*aac(6*′*)-Ib-cr*	*bla*_CTX-M-65_/*bla*_TEM_	
SHI 3G355	2	>64	>8	2	4	D87N	WT	*aac(6*′*)-Ib-cr*	*bla*_TEM_	
SHI 3G769	2	>64	>8	2	4	D87N	WT	*aac(6*′*)-Ib-cr*	*bla*_CTX-M-14_	
SHI 3G938	16	>64	>8	2	4	D87N	WT		*bla*_OXA_*/bla*_CTX-M-55_	
SHI 3G939	16	>64	>8	2	4	D87N	WT		*bla*_OXA_*/bla*_CTX-M-55_	
SH13G1219	8	>64	>8	>4	128	D87Y	WT	*aac(6*′*)-Ib-cr*	*bla*_CTX-M-15_*/bla*_TEM_	*mphA*
SHI 3G1825	2	>64	>8	>4	128	D87Y	WT	*aac(6*′*)-Ib-cr*	*bla*_CTX-M-15_*/bla*_TEM_	*mphA*
SHI 4G287	4	>64	>8	2	8	WT	WT	*aac(6*′*)-Ib-cr*	*bla*_OXA_*/bla*_CTX-M-125_	
SHI 4G294	8	64	>8	2	8	D87N	WT	*aac(6*′*)-Ib-cr*	*bla*_OXA_*/bla*_CTX-M-125_	
SHI 4G300	4	64	>8	2	64	D87N	WT	*aac(6*′*)-Ib-cr*	*bla*_OXA_*/bla*_CTX-M-125_	
SHI 4G1345	4	>64	>8	2	4	D87Y	WT		*bla*_OXA_*/bla*_CTX-M-55_*/bla*_TEM_	
SH14G1601	2	>64	>8	2	4	D87N	WT	*aac(6*′*)-Ib-cr*	*bla*_CTX-M-55_	

*FOX, cefoxitin; CRO, ceftriaxone; XNL, ceftiofur; CIP, ciprofloxacin; AZI, azithromycin; WT, wild type.*

## Discussion

In present study, we found that *S.* Typhimurium isolates in Shanghai, China, exhibited high rates of resistance to traditional antimicrobials, such as tetracycline, ampicillin, nalidixic acid, and sulfisoxazole. Moreover, resistance to cephalosporins, ciprofloxacin and azithromycin were found in 20.6, 3.6, and 5.5% of the isolates, respectively. Contradictory to this result from the current study, the report of the NARMS indicated that the number of cephalosporin-resistant, ciprofloxacin-resistant and azithromycin-resistant isolates was lower than 6, 1, and 1% (2011–2014), respectively (Centers for Disease Control and Prevention, 2016). More importantly, 84.6% of the isolates were found to exhibit MDR in our study, which is significantly higher than the percentage observed from the surveillance data during 2005 to 2010 in Shanghai and that indicated in the NARMS report for the same period (*P* < 0.05) (Zhang et al., 2014; Centers for Disease Control and Prevention, 2016). Notably, in the current study, we found that 22 MDR isolates with decreased susceptibility to ciprofloxacin were co-resistant to cephalosporins. Six of the 22 isolates also showed resistance to azithromycin. This phenomenon has only been reported previously in two clinical *S.* Typhimurium strains in China (Wong et al., 2014). If these MDR strains are as prevalent as *S.* Typhimurium DT104 all over the world, it will pose a great threat to global public health. Our findings indicate that it is therefore necessary to continue monitoring the antimicrobial resistance of *S.* Typhimurium isolates to help determine the appropriate antimicrobial therapy for patients with *S.* Typhimurium infection. More importantly, it is necessary to study the mechanisms underlying the antimicrobial resistance in these isolates.

The main mechanisms of quinolone-resistance in *Salmonella* have been attributed to several point mutations in the QRDRs of the *gyrA* and *parC* genes and the PMQR genes (Hopkins et al., 2007; Kim et al., 2013). Mutations at different sites contribute to different levels of resistance to ciprofloxacin, and simultaneous mutations in both *gyrA* and *parC* genes produce high levels of ciprofloxacin resistance. Besides, most isolates with decreased susceptibility to ciprofloxacin contain a mutation in *gyrA* or PMQR genes (Ling et al., 2003; Lin et al., 2015). In the present study, we found that six isolates had double mutations (S83F/D87N or D87G) in the *gyrA* gene and a single mutation (S80R) in the *parC* gene. Additionally, one isolate with four mutations, including two in *gyrA* gene (S83F/D87N) and two in *parC* gene (T57S/S80R). These mutations have been previously reported to be most common in ciprofloxacin-resistant *S.* Typhimurium strains (Wasyl et al., 2014). PMQR was initially identified in *Klebsiella pneumonia* in 1998 (Martinez-Martinez et al., 1998), since then, various types of PMQR genes have been detected in *Salmonella* all over the world (Cattoir et al., 2007; Cavaco et al., 2009; Kim et al., 2013). Previous studies have shown that the presence of *qnr* genes increase the MICs for ciprofloxacin by 8–16-fold (Kim et al., 2013). In addition, the presence of *aac(6′)-Ib-cr* genes can increase the selection of chromosomal mutants that cause ciprofloxacin resistance (Robicsek et al., 2006). In the current study, we also showed that the existence of PMQR genes remarkably enhances the MICs (≥2 μg/mL) of ciprofloxacin in the presence of a single or no point mutation in the *gyrA* gene in *S.* Typhimurium isolates. The emergence of PMQR genes described in this report has also been frequently reported previously in *Salmonella* isolates in other parts of China (Lu et al., 2011; Jiang et al., 2014; Wong et al., 2014), which suggests that the PMQR genes have been prevalent in China.

Since the discovery of *bla*_CTX-M_ genes in Japan, Europe, and South America in the 1980s (Matsumoto et al., 1988; Bauernfeind et al., 1990, 1992), they have quickly replaced *bla*_TEM_ and *bla*_SHV_ as the major ESBL (D’Andrea et al., 2013). In the present study, of the 115 cephalosporin-resistant isolates, 62.5% harbored *bla*_CTX-M_ genes, in which *bla*_CTX-M-55_ was the most common gene, followed by *bla*_CTX-M-14_. This phenomenon proved that the *bla*_CTX-M-1_ and *bla*_CTX-M-9_ groups are popular types in *S.* Typhimurium isolates in Shanghai. Besides, the subtypes of *bla*_CTX-M-123_ and *bla*_CTX-M-125_ have also been identified in *E. coli* (Rao et al., 2014), but have not been found in *Salmonella*. To the best of our knowledge, this study is the first to report *bla*_CTX-M-123_ and *bla*_CTX-M-125_ in *S.* Typhimurium. In addition, 52 (45.2%), 43 (37.4%), and 3 (2.6%) isolates were found to contain the *bla*_TEM-1_, *bla*_OXA-1_, and *bla*_SHV -12_ genes, respectively. Because the ESBL genes are usually located on the antimicrobial-resistance plasmid of the bacteria, they can be easily disseminated between different species of bacteria with the transfer of the drug-resistance plasmid (Arlet et al., 2006).

As the number of cephalosporin-resistant and ciprofloxacin-resistant *Salmonella* strains gradually increased, an alternative antimicrobial class was needed to manage *Salmonella* infections. Azithromycin showed excellent ability to accumulating at high intracellular concentrations, and it achieved intracellular concentrations of 50 to 100 times greater than the serum levels (Panteix et al., 1993). Therefore, azithromycin was recommended for the treatment of invasive *Salmonella* infections by the FAD (Sjolund-Karlsson et al., 2011). However, in the present study, 31(5.5%) isolates were found to be resistant to azithromycin. Previously, *Salmonella* strains resistant to azithromycin have also been found in other countries (Sjolund-Karlsson et al., 2011; Kalonji et al., 2015). The plasmid-borne *mphA* gene was reported as one of the reasons of azithromycin-resistance (Glynn et al., 1998). In the present study as well, 15 high-level azithromycin-resistant isolates (MIC ≥ 128 μg/mL) harbored the *mphA* gene, while other 16 (MIC: 16–64 μg/mL) did not. All azithromycin-resistant isolates were negative for *mphB, ermA, ermB, ereA, mefA*, and *msrA*. These results indicate that the *mphA* gene may mediate a high level of resistance to azithromycin in *Salmonella*, as described previously in *Shigella* in China (Zhang et al., 2016). In addition, azithromycin resistance may arise from other possible mechanisms, such as mutations in the *rlpD* and *rlpV* genes (Roberts, 2008). At present, few studies have been focused on investigating azithromycin resistance mechanisms in *Salmonella*. The findings of the present study and those of other previous studies emphasize the need for investigating the further mechanisms underlying azithromycin resistance in *Salmonella* isolates.

Antimicrobial-resistance genes are usually located on plasmids and therefore can be easily disseminated; for example the ESBL genes often coexist with the genes encoding resistance to other antimicrobial agents in plasmids, which can easily lead to a MDR phenotype in ESBL-producing bacteria (Jiang et al., 2014). In this study, we found that 15 isolates showed both ESBL and PMQR genes, in which concurrently displayed decreased susceptibility to cephalosporins and ciprofloxacin. Besides, 2 of the 15 isolates co-contained *mphA* genes, which were resistant to azithromycin.

## Conclusion

In this study, we reported the common occurrence of MDR in *S.* Typhimurium isolates in Shanghai, China. We found that resistance to ciprofloxacin, cephalosporins, and azithromycin was most prevalent among the isolates. Among the MDR isolates, we found various transferrable antimicrobial-resistance genes, which included ESBL, PMQR and *mphA* genes, and some isolates even contained at least two types of these genes. The dissemination of these genes poses a huge threat to the control of *S.* Typhimurium infection in the world. Our findings indicate that it is imperative to continue monitoring the prevalence of the above-mentioned three types of antimicrobial resistance and their resistant genes in *S.* Typhimurium isolates. Future studies should be focused on identifying ways to prevent the dissemination of these antimicrobial-resistance genes.

## Author Contributions

SQ, HS, and JW designed the study, XX participated in the collection of the samples. BL, FW, XY, CS, XH, and JX completed identification and preservation of samples, YL, XD, HaL, and JW were responsible for the experiments. JW, QM, HoL, RH, and CY analyzed the data. JW wrote the manuscript, and SQ and HS provided academic revision for the manuscript. All the authors have read and approved the final draft of the manuscript.

## Conflict of Interest Statement

The authors declare that the research was conducted in the absence of any commercial or financial relationships that could be construed as a potential conflict of interest.
